# Neuromuscular Fatigue and Perceived Fatigability in the Hours Following a High‐Intensity Endurance Running Depend on the Exercise Protocol

**DOI:** 10.1002/ejsc.70176

**Published:** 2026-07-27

**Authors:** Yago Medeiros Dutra, Paloma Tavares Mendonça, Stuart Goodall, Alessandro Moura Zagatto

**Affiliations:** ^1^ Laboratory of Physiology and Sport Performance (LAFIDE) School of Sciences São Paulo State University (UNESP) Bauru Brazil; ^2^ Department of Sport, Exercise, & Rehabilitation Faculty of Health and Life Sciences Northumbria University Newcastle Upon Tyne UK; ^3^ Faculty of Health Sciences Physical Activity Sport and Recreation Research Focus Area North‐West University Potchefstroom South Africa

**Keywords:** exercise‐induced fatigue, HIIT, post‐exercise recovery, twitch interpolation

## Abstract

This study investigated neuromuscular fatigue and perceived fatigability across different high‐intensity endurance running protocols. Twelve males (mean ± SD; $\dot{V}O_{2\;\max}$: 52.2 ± 6.9 mL/kg/min) ran ∼8.0 km: (i) at the velocity associated to the respiratory compensation point (vRCP; 5 × 8 min bouts ‒ RUN_100%RCP_); (ii) at 30% above vRCP (10 × 3 min bouts ‒ RUN_130%RCP_); and (iii) at 50% above vRCP (20 × 80 s bouts ‒ RUN_150%RCP_). Before and for 6 h post‐exercise, voluntary and evoked contractions of knee‐extensors were assessed, alongside tiredness, leg pain, and perceived recovery. Following all protocols, voluntary activation remained depressed for up to 4 h post‐exercise, while voluntary peak force was reduced for at least 6 h (*p* < 0.05). Other neuromuscular function markers reduced (*p* < 0.05) according to the protocol. No time × protocol interaction was observed for any neuromuscular function marker (*p* > 0.05). Aggregate data (protocol effect, *p* < 0.05) evidenced greater reduction in neural drive to *vastus lateralis* (RMS_VL_/Mw_amp_) after RUN_100%RCP_ (−20%) compared to RUN_130%RCP_ (−3%) and RUN_150%RCP_ (−7%); and greater reduction in late rate of force development (RFD; 100–200 ms) and contractile function (Qtw_pot_) after RUN_150%RCP_ (−18% and −8%, respectively) than RUN_100%RCP_ (−8% and −2%, respectively). Leg pain and tiredness remained elevated (*p* < 0.05) for at least 6 h following all protocols. Leg pain and tiredness were greater for up to 4 h and for at least 6 h, respectively, after RUN_150%RCP_ than RUN_100%RCP_ (time × protocol interaction; *p* < 0.05). The choice of exercise protocol influences the extent of reduction in *vastus lateralis* neural drive, impairments in knee‐extensors contractile function, and perceived fatigability for hours following a high‐intensity endurance running.

## Highlights

- High‐intensity endurance running (8.1 ± 0.6 km) elicits knee‐extensor fatigue and perceived fatigability requiring at least 6 h to resolve.
- The aggregate reduction in neural drive to *vastus lateralis* (RMS_VL_/Mw_amp_) in the following 6 h post‐exercise (i.e., main effect of protocol) was greater after running at the velocity associated with the respiratory compensation point (vRCP; 5 × 8 min bouts) compared to running at 30% above vRCP (10 × 3 min bouts) and at 50% above vRCP (20 × 80 s bouts).
- The aggregate reduction in knee‐extensors contractile function (RFD_100–200 ms_ and Qtw_pot_) in the following 6 h post‐exercise (i.e., main effect of protocol) was greater after running at 50% above vRCP (20 × 80 s bouts) compared to running at vRCP (5 × 8 min bouts). This was accompanied by greater leg pain and tiredness for up to 4 h and for at least 6 h post‐exercise, respectively.
- To the best of our knowledge, this is the first study to demonstrate that the extent of reduction in *vastus lateralis* neural drive, impairments in knee‐extensors contractile function, and perceived fatigability in the hours following a high‐intensity endurance running depend on the exercise protocol undertaken.

## 1 Introduction

High‐intensity endurance running is a key component of training across many individual and team sports (Buchheit and Laursen 2013a; MacInnis and Gibala 2017). The metabolic and mechanical stresses imposed by high‐intensity endurance running lead to fatigability (Behrens et al. 2023; Enoka and Duchateau 2016). This is defined as a transient state characterized by a decline in force output (performance fatigability) and psychophysiological changes, including pain in exercised muscles and tiredness sensation (perceived fatigability) (Behrens et al. 2023; Enoka and Duchateau 2016). Performance fatigability is further characterized by underlying mechanisms, which may include central factors (e.g., reduced neural drive and impaired voluntary activation) and peripheral factors (e.g., changes in muscle contractile function, loss of force production during a muscle twitch in response to high‐frequency and low‐ vs. high‐frequency muscle stimulation) (Behrens et al. 2023; Enoka and Duchateau 2016). Although high‐intensity endurance running is widely practiced, the mechanisms of fatigability in locomotor efforts have been mostly investigated using cycle‐based exercises (Enoka and Duchateau 2016). The applicability of these findings to running is questionable, due to fundamental differences in metabolic and neuromuscular demands between these two modalities of exercise (Bijker et al. 2002).

For instance, the force production during the concentric phase of cycling has a greater reliance on knee‐extensors contractile elements (Bijker et al. 2002), which leads to greater impairments in contractile function compared to running (Brownstein et al. 2022; Lepers et al. 2002; Place et al. 2004). These impairments are exacerbated when cycling at intensities far above the maximum metabolic steady state (MMSS) (Iannetta et al. 2022; Thomas et al. 2016, 2015), as this leads to pronounced metabolic disturbances within the muscles (e.g., increases in [Pi], [H^+^], [K^+^], [La^−^]) (Chidnok et al. 2013; Pollak et al. 2014; Zagatto et al. 2025). In contrast, force production in running has a greater dependence on the stretch‐shortening cycle (SSC) and the re‐use of stored elastic energy (Bijker et al. 2002). Therefore, the extent of metabolic disturbances and contractile function impairments in knee‐extensors following cycling is unlikely to be directly transferable to running. This is supported by evidence that quadriceps' delta efficiency (defined as the ratio of increment in external power output to the increase in metabolic power required to produce it) is greater in running than in cycling (Bijker et al. 2002). This suggests that the metabolic effects of increasing intensity are likely to be less severe in the former. Furthermore, while SSCs improve contractile efficiency (Bijker et al. 2002), they can also induce muscle microtrauma (Stocchero et al. 2014; Wiewelhove et al. 2015) and reduce the facilitation of Ia afferents onto motoneurons (Avela et al. 1999). These effects contribute to the greater impairments in muscle activation after running compared to cycling, regardless of exercise intensity (Brownstein et al. 2022; Lepers et al. 2002; Place et al. 2004). While research suggests that exercise intensity affects muscle activation impairments following cycling (Thomas et al. 2016, 2015), its influence on muscle activation following running remains unknown.

To our knowledge, only (Skof and Strojnik 2006a, 2006b) has investigated the mechanisms and recovery time‐course of fatigability after high‐intensity endurance running. These authors observed impaired knee‐extensors peak force (during maximal voluntary contractions; MVCs) for up 60 min after a continuous 6‐km track run performed at a velocity corresponding to the onset of blood lactate accumulation (Skof and Strojnik 2006a). However, no such impairment was observed following 5 × 300 m track runs at a velocity 5% below all‐out 400 m run (Skof and Strojnik 2006b). Depression in force evoked by low‐frequency stimulation (referred as prolonged low‐frequency force depression) (Allen et al. 2008) persisted for up to 60 min following the 6 km track run (Skof and Strojnik 2006a), whereas both the decrease in evoked force from a single stimulus and from a low‐frequency stimulation completely recovered within 20‐min after the 5 × 300 m track runs (Skof and Strojnik 2006b). Notably, neither running protocol induced significant changes in voluntary activation capacity, as assessed by the twitch interpolation technique (Skof and Strojnik 2006a, 2006b). Although the studies of (Skof and Strojnik 2006a, 2006b) provide valuable insights, a direct comparison of neuromuscular function impairments following high‐intensity endurance running protocols has not yet been performed. Furthermore, the perceived fatigability responses following different running protocols remain unexplored. This is an important gap in our knowledge, as perceived fatigability can impair performance and contribute to long‐term health risks (Kellmann et al. 2018).

Accordingly, the aim of this study was to evaluate neuromuscular fatigue and perceived fatigability following distinct protocols of high‐intensity endurance running. Fatigability dynamics were assessed across running protocols matched for volume (i.e., distance covered in kilometers), but performed at the (i) velocity associated with the respiratory compensation point (vRCP), (ii) 30%, and (iii) 50% above vRCP. The vRCP was used as a surrogate marker of MMSS for training prescription (Keir et al. 2022). These intensity levels were selected to align with exercise protocols common in conditioning programs (MacInnis and Gibala 2017; Casado et al. 2022), and to investigate neuromuscular fatigue and perceived fatigability across a broad spectrum of physiological stress in running. Specifically, the run at vRCP was designed to mimic a high‐intensity continuous endurance training session, also termed tempo run, which elicits a slower rate of metabolite accumulation and lower neuromuscular demand than higher intensities (Iannetta et al. 2022; Casado et al. 2022). The run at 30% above vRCP was adopted to simulate a high‐intensity intermittent training session with long work durations (Buchheit and Laursen 2013a). This protocol is typically used to increase time accumulated in the “red zone” (i.e., an intensity exciding 90% of maximal oxygen uptake) (Buchheit and Laursen 2013a), while reducing metabolic disturbance and neuromuscular load (Buchheit and Laursen 2013b; Vuorimaa et al. 1996; Buchheit et al. 2012). Finally, the run at 50% above vRCP was designed to be a high‐intensity intermittent training session that induces substantial metabolite accumulation and high neuromuscular demand (Buchheit and Laursen 2013a, 2013b), analogous to the responses elicited by sprint interval training (Skof and Strojnik 2006b; Tomazin et al. 2012). Investigating fatigability across protocols with such distinct demands addresses a notable gap in the literature, as varied running protocols are common in training and competitive routines, but their specific neuromuscular fatigue and perceived responses lack further characterization. Furthermore, since athletes' schedules may involve multiple daily running sessions to increase overall training volume (Tønnessen et al. 2014) or combine it with complementary practices (e.g., sport‐specific technical‐tactical exercises) (Sparkes et al. 2020), we conducted a 6 h post‐exercise monitoring period. This approach may offer insights into the proper management of fatigability within a training/competition day (Kellmann et al. 2018). We hypothesized that characteristics and the extent of fatigability responses would be dependent on the running protocol. Specifically, running at 50% above vRCP is hypothesized to cause greater impairments in contractile function and leg pain compared to lower‐intensities protocols. In contrast, impairments in muscle activation capacity and tiredness are expected to be more pronounced following running at vRCP compared to the higher‐intensity protocols.

## 2 Materials and Methods

### 2.1 Participants and Ethics Approval

A minimum of 10 participants was estimated to achieve a power of 0.90 in *f* tests, setting an alpha of 0.05, based upon Azevedo et al. (2022). These authors observed distinct levels of muscle contractile function disturbance after cycling to task failure at different intensities (i.e., distinct levels of reduction in Qtw_pot_ of knee extensors; partial eta squared = 0.25). Additionally, due to the known sex‐related differences on performance fatigability (Azevedo et al. 2022), we recruited males only to limit heterogeneity within the data. Thus, 15 healthy, recreational runners (training volume of 15–40 km/week) were recruited to participate of the study. A 50% attrition rate was adopted to account for the high dropout rate observed in previous studies of our research group with similar procedures. Participants were included if they were not under medical or nutritional treatments in the 6 months before the study, and if they had no known neurological, cardiovascular, or musculoskeletal disorders that could potentially affect the evaluated outcomes of the investigation. After being made aware of the experimental procedures, all participants provided written informed consent. Due to successive absences in the trials (*n* = 3), the final sample consisted of 12 participants (means ± SD: age 24 ± 5 years old; height 175.7 ± 6.6 cm; body mass 71.7 ± 8.9 kg; training volume, 23.3 ± 6.5 km covered/week). Institutional ethical approval was provided for this study (Number 50075421.9.0000.5398), and all procedures were conducted in accordance with the Declaration of Helsinki.

### 2.2 Experimental Design

**FIGURE 1:**
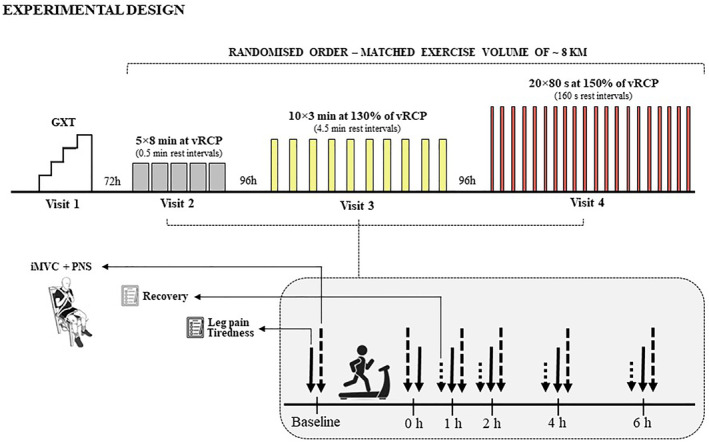
Schematic representation of the investigation design. GXT, graded exercise test to assess cardiorespiratory function; vRCP, velocity associated with the respiratory compensation point; iMVC, maximal voluntary isometric contraction of knee‐extensors (dominant lower‐limb, self‐reported); PNS, peripheral electrical stimulation; *N* = 12.

The experimental design of the study is presented in Figure 1. Participants visited the laboratory on 4 occasions with a 72–96 h interval between visits. During visit 1, participant characteristics were recorded (i.e., stature and body mass), and a graded exercise test (GXT) was performed. Participants were also familiarized with the experimental procedures of the study (i.e., questionnaires, scales, maximal knee‐extensors contractions, and peripheral electrical stimulation). During visits 2, 3, and 4, participants performed three different running protocols, in a randomized order: a continuous run at a velocity corresponding to the respiratory compensation point (RUN_100%RCP_); a high‐intensity interval run 30% above vRCP (RUN_130%RCP_); and a high‐intensity interval run 50% above vRCP (RUN_150%RCP_). See below for further details. Despite differences in exercise protocol, each participant performed the same volume in each running protocol.

Throughout RUN_100%RCP_, RUN_130%RCP_, and RUN_150%RCP_, rate of pulmonary oxygen consumption ($\dot{V}O_{2}$) and ventilation (${\dot{V}}_{E}$) were recorded. At every 20% of exercise completion, earlobe blood samples were collected to measure blood lactate concentrations ([La^−^]). In the last 30 s of these same time‐windows, ratings of perceived exertion (RPE), pain in lower limbs (leg pain), and the sensation of tiredness (tiredness) were collected. At baseline, immediately (within ∼1 min) after each exercise and 1, 2, 4, and 6 h post‐exercise, neuromuscular assessments (consisting of maximal isometric contractions of knee‐extensors [iMVC] with peripheral electrical stimulation [PNS]) were performed. Rating of leg pain and tiredness was collected at the same time points, and perception of recovery was also collected 1, 2, 4, and 6 h post‐exercise. All exercises (i.e., GXT, RUN_100%RCP_, RUN_130%RCP_, and RUN_150%RCP_) were performed on a motorized treadmill (ATL, Inbramed, Brazil).

Prior to each visit, all participants were instructed to avoid consumption of food and caffeinated beverages for at least 2 and 8 h, respectively, and to abstain from vigorous physical activity for 24 h. Before visit two (i.e., first experimental trial), participants were instructed to carry out a dietary recall and to repeat the same eating pattern prior to remaining visits. Upon arrival at all experimental visits, participants were required to rate their overall perception of sleep quality on a visual scale (0‐10; 0‐terrible to 10‐excellent) (Snyder et al. 2018). If sleep quality was rated below 6 (fair), the participants were released, and the visit was rescheduled (this occurred on 3 occasions). After responses were recorded immediately post‐exercise, participants consumed cereal bars (Nutry, Paraná, Brazil) and processed juices (Maguary, São Paulo, Brazil), aiming to ingest 1.1 g/kg of body mass/hour of carbohydrate, which has been recommended to maximize glycogen repletion (McCartney et al. 2018). Water was consumed *ad libitum*, and the amount of food and water ingested post‐exercise during visit 2 was recorded and repeated in visits 3 and 4. All participants performed experimental visits at the same time of day (± 2 h), and all tests were performed in an environmentally controlled room (temperature: 20°C–22°C; humidity 40%–60%). During the 6 h following the run trials, all participants waited for post‐exercise assessments in a quiet, temperature‐controlled room (20°C–22°C; 40%–60% humidity) inside the laboratory. During this period, participants were permitted to engage in personal activities (e.g., using smartphones, laptops, or books for work/study) and use the restroom, but were prohibited from engaging in physical activity, napping, or showering.

#### 2.2.1 Exercise Protocols

##### 2.2.1.1 Graded Exercise Test

The GXT was performed to determine the maximal rate of oxygen consumption ($\dot{V}O_{2\;\max}$), RCP, and associated velocities (i.e., v$\dot{V}O_{2\;\max}$ and vRCP, respectively). The vRCP was used to set the intensity of running during experimental visits (i.e., RUN_100%RCP_, RUN_130%RCP_, and RUN_150%RCP_). The GXT started at 8 km/h before the velocity was increased by 1.5 km/h every 2 min until task failure (Dutra et al. 2025). Five minutes after the end of the GXT, participants performed a constant work rate exercise until task failure at a velocity 5% greater than the highest velocity achieved during GXT (Dutra et al. 2025). This effort was performed to add confidence in the establishment of a $\dot{V}O_{2}$ response more likely to reflect the true $\dot{V}O_{2\;\max}$ (Iannetta et al. 2020). In both cases, task failure was determined when participants disengaged from the effort despite the use of strong verbal encouragement.

##### 2.2.1.2 Different running protocols: RUN_100%RCP_, RUN_130%RCP,_ and RUN_150%RCP_

RUN_100%RCP_ consisted of 5 × 8 min bouts at vRCP separated by 0.5 min intervals. This protocol was designed to simulate a high‐intensity continuous endurance run (also called tempo run) (Casado et al. 2022), with brief pauses incorporated solely for earlobe blood collection (see below for details). RUN_130%RCP_ consisted of 10 × 3 min bouts at 30% above vRCP separated by 4.5 min passive rest; and RUN_150%RCP_ was composed of 20 × 80 s bouts at 50% above vRCP separated by 160 s passive rest. The duration of the bouts in RUN_130%RCP_ and RUN_150%RCP_ was chosen aiming to match the estimated distance covered by the participants between RUN_100%RCP_, RUN_130%RCP_, and RUN_150%RCP_ (i.e., distance in km = velocity in km/h × exercise time in hours). For example, with a vRCP of 10 km/h, after 40 min (the usual duration of this type of training session) (MacInnis and Gibala 2017), the participant would be expected to cover ∼6.7 km. To cover this same distance at 13 km/h (i.e., 130% of vRCP) and 15 km/h (i.e., 150% of vRCP), the participant would take ∼31 min (i.e., ∼10 × 3 min) and ∼26 min (i.e., ∼20 × 1 min and 20 s). These intensity levels were selected in accordance with exercise protocols commonly used in conditioning programs (MacInnis and Gibala 2017). The recovery intervals for the RUN_130%RCP_ and RUN_150%RCP_ protocols were selected to produce distinct work/relief, thereby inducing distinct levels of metabolic and neuromuscular disturbance. Specifically, a 4.5 min pause in RUN_130%RCP_ established a work/relief ratio of 1.5. In intermittent protocols with extended work durations (> 3 min) and intensities near 95% of v$\dot{V}O_{2\;\max}$, this ratio is known to reduce both the anaerobic glycolytic energy contribution to exercise and the demand on the neuromuscular system (Buchheit and Laursen 2013a, 2013b). For the RUN_150%RCP_, a 160 s pause created a work/relief ratio of 2. This higher ratio is associated with greater metabolic disturbance and neuromuscular demand in intermittent protocols performed at intensities ranging from above v$\dot{V}O_{2\;\max}$ to *all‐out* (Buchheit and Laursen 2013a, 2013b). All participants were able to complete the proposed running protocols.

### 2.3 Data Collection and Analysis

#### 2.3.1 Respiratory Data

Breath‐by‐breath pulmonary gas exchange and ${\dot{V}}_{E}$ were continuously measured using a metabolic cart (Quark, CPET, COSMED, Rome, Italy) during GXT, RUN_100%RCP_, RUN_130%RCP_, and RUN_150%RCP_. The metabolic cart was calibrated before each test according to the recommendations from the manufacturer. Breath‐by‐breath data were examined to exclude aberrant breaths, values varying more than ± 3 SD from the local mean were removed (Keir et al. 2014). The breath‐by‐breath data were subsequently interpolated every 1 s (Keir et al. 2014) using appropriate software (OriginPro v8.0, Origin Lab Corporation, Massachusetts, United States). The highest $\dot{V}O_{2}$ average from the last 20 s of each stage was determined as $\dot{V}O_{2\;\max}$ when it was similar (± 2.1 mL/kg/min) to $\dot{V}O_{2}$ average from the last 10 s recorded in the constant work‐rate exercise carried out 5 min after GXT (Keir et al. 2014). The minimal exercise velocity at which $\dot{V}O_{2\;\max}$ was reached was recorded as v$\dot{V}O_{2\;\max}$ (Billat et al. 1999). For the estimation of RCP, raw profiles of ${\dot{V}}_{E}$, carbon dioxide output ($\dot{V}{\text{CO}}_{2}$), their ratio (i.e., ${\dot{V}}_{E}/\dot{V}{\text{CO}}_{2}$), respiratory exchange ratio (RER), and end‐tidal pressure of CO_2_ (PetCO_2_) were plotted against time. RCP was identified as the point at which PetCO_2_ began a precipitous fall after a period of isocapnia concomitantly with a second and first breakpoint in the ${\dot{V}}_{E}$ and ${\dot{V}}_{E}/\dot{V}{\text{CO}}_{2}$ relationships, respectively (Keir et al. 2022). The vRCP was identified by the projection of RCP (time at which it occurs) at GXT stages, and where necessary, v$\dot{V}O_{2\;\max}$ and vRCP were adjusted according to (Kuipers et al. 1985). The test protocols used in this study did not account for the mean response time of respiratory $\dot{V}O_{2}$ or the non‐linear relationship between work‐rate and $\dot{V}O_{2}$ above lactate threshold (Keir et al. 2014). Therefore, the vRCP reported may reflect a work rate exceeding the maximum metabolic steady state (Keir et al. 2014). During RUN_100%RCP_, RUN_130%RCP_, and RUN_150%RCP_, the $\dot{V}O_{2}$ and ${\dot{V}}_{E}$ average of the last 20 s of each time window corresponding to 20% of exercise completion was calculated. These responses were analyzed and reported using *raw* data.

#### 2.3.2 Neuromuscular Function

##### 2.3.2.1 Maximal Isometric Voluntary Contraction, EMG Activity, and Peripheral Nerve Stimulation

The iMVCs were performed with the dominant knee‐extensors (self‐reported). For the iMVCs, participants were positioned on a custom‐made chair designed to keep the hips and knees flexed at 90°. Participants were firmly secured with straps fastening legs, hips, and chest. For the assessment of neuromuscular function immediately after exercise, it took around 1 min (57 ± 9 s), to maneuver the participants from the treadmill to the isometric set up in preparation to start the test (e.g., electrode and wire placement, ankle attachment, signal stabilization). Except in the assessments carried out immediately after exercise, three iMVCs interspersed by 30 s and followed by 2‐min rest, were performed before all trials as a warm‐up procedure. The peak force achieved during these warm‐up iMVCs was used to ensure that participants reached their peak force during neuromuscular assessment.

Force signals were acquired using a calibrated load cell with a 0.98‐N resolution (MK Controle, Brazil). The load cell was attached to the ankle by a metal rod immediately above the region of the medial malleolus using straps with heavy duty Velcro. The EMG signal from *vastus lateralis* was acquired by a pair of self‐adhesive Ag/AgCl surface electrodes (Meditrace 100, Covidien, USA) placed on the 1/3 distal of muscle and on patella (ground electrode). The signal was amplified by an octal bio‐amplifier (FE136, ADInstruments, New Zealand). Before electrode placement, the skin sites were shaved, lightly abraded using a sandpaper, and cleaned with alcohol. Both muscular force and EMG signals were analogue‐to‐digitally converted by PowerLab system (16/35, ADInstruments, New Zealand) and analyzed offline using the LabChart 8 software (ADInstruments, New Zealand).

Transcutaneous electrical stimulations were delivered over the femoral nerve (pulse duration: 1 ms; square wave) using a high‐voltage electrical stimulator (Bioestimulador V2 400 V peak‐to‐peak; Insight, Brazil). Stimulations were delivered through Ag/AgCl electrodes (10 mm of diameter, Kendall Medi‐Trace, USA) placed over the femoral triangle (cathode) and through carbon rubber electrodes (32.5 mm of diameter, CARCI, Brazil) placed over the femoral greater trochanter (anode). The position of the EMG and stimulation electrodes was marked with indelible ink, being replaced during the trials when necessary. Doublet high‐frequency stimulations (i.e., 100 Hz) were delivered during the plateau of the iMVC effort (∼3 s after iMVC start) as well as within 3 s after the iMVC at rest. Single and doublet low‐frequency (10 Hz) stimulations were delivered to the relaxed muscle at 7 and 10 s after iMVC, respectively. The magnitude of the electrical stimuli was determined individually in each visit by the application of consecutive incremental single stimuli of 30 mA to the relaxed muscle (starting at 30 mA) until reaching the involuntary force plateau. The force plateau was reached with 280 ± 58 mA. The intensity used throughout the trials corresponded to a value 30% higher than this to ensure supramaximal intensity (Dutra et al. 2023).

The peak voluntary force (iMVC_Force_) was defined as the highest voluntary force achieved within a 100 ms period during the iMVC. In all cases, iMVC_Force_ occurred prior to stimulation. Early‐ and late‐phase rate of force development (RFD) during the MVC was also calculated as the slope of the force versus time curve (Δforce/Δtime). Early and late RFD were determined from 0 to 50 ms (RFD_0–50 ms_) and from 100 to 200 ms (RFD_100–200 ms_) relative to the onset of MVC, respectively. The onset of the MVC was manually detected by the same investigator as the last depression before force deflects above the range of the baseline noise, keeping a fixed scale across all analyses (200 ms vs. 0.5 N) (Maffiuletti et al. 2016). To assess VA, superimposed force amplitude elicited by high‐frequency doublet stimuli during iMVC and at rest after iMVC (Db100_F_) were recorded. VA was calculated as: VA (%) = [1‐(superimposed force) × (force level at stimulation/iMVCForce)/(Db_100F_)] × 100 (Strojnik and Komi 1998). Contractile properties were evaluated by recording the peak force evoked by the single stimulus (Qtw_pot_), high‐frequency doublet stimuli (Db100_F_), and the low‐to‐high frequency doublet stimuli (Db10_F_/Db100_F_, respectively). The EMG signal was band‐pass filtered (20–500 Hz), and the root mean square from the signal 0.5 s around the iMVC_Force_ was calculated (RMS_VL_). When the RMS_VL_ covered the superimposed contraction stimulus, the 0.5 s time was shifted to the left until the stimulus artifact noise in the EMG signal was avoided. The amplitude of EMG response evoked by the single stimulus was recorded (Mw_amp_) and the ratio between RMS_VL_ and M‐wave amplitude was calculated (RMS_VL_/Mw_amp_). The iMVC_Force_ was used as global neuromuscular function marker; VA indicated voluntary activation level, while RMS_VL_/Mw_amp_ demonstrated neural drive to *vastus lateralis* (i.e., propagation of electrical signals from the central nervous system to the muscle). The Qtw_pot_, Db100_F,_ and Db10_F_/Db100_F_ reflected contractile function capacity, force production in response to high‐frequency muscle stimulation, and force production in responses to low‐versus high‐frequency muscle stimulation, respectively; and Mw_amp_ indicated sarcolemma's capacity to propagate action potentials (Millet et al. 2011). All neuromuscular function data were expressed as percentage change from baseline.

#### 2.3.3 Perceptual Responses

RPE was assessed using the 6‐20 top‐to‐bottom Borg's scale (G. A. Borg 1982); leg pain was assessed by 0–10 top‐to‐bottom Borg's scale (E. Borg et al. 2010); and tiredness was assessed by 10‐0 top‐to‐bottom Micklewright's scale (Micklewright et al. 2017). Participants' perception of recovery was assessed using the Total Quality Recovery scale proposed by Kentta and Hassmen (6‐20 top‐to‐bottom scale, “very, very poor recovery” to “very, very good recovery” (Kenttä and Hassmén 1998). Participants were inquired to rate their current recovery status in relation of the end of the exercise and, before answering, they were asked to direct their attention to signs such as sleepiness, mood states, soreness, or heaviness in their legs. All perceptual measures were reported and analyzed using *raw* data.

#### 2.3.4 Blood Lactate Concentration During Exercise

Blood samples from the earlobe were collected following each 20% of exercise completion during RUN_100%RCP_, RUN_130%RCP_, and RUN_150%RCP_ to measure [La^−^]. Blood samples (25 *μ*L) were collected in heparinized micro‐hematocrit tubes, immediately deposited into polyproline microtubes containing 50 *μ*L of sodium fluoride at 1%, stored at −20° Celsius and then analyzed in a biochemical analyzer YSI 2900 (Yellow Spring Instruments, Ohio, USA). The equipment error is reported to be ± 2%, according to the manufacturer's information. The [La^−^] was reported using *raw* data.

### 2.4 Statistical Analysis

All data are presented as mean ± SD. The SPSS (v20.0 IBM Corp., Armonk, NY) was used for all statistical analyses, with statistical significance set at *α* of 0.05. Normality of data was verified using the Shapiro‐Wilk test. One‐way repeated‐measure ANOVA was used to assess the effect of time (0%–100% of exercise completion; baseline to 6 h post‐exercise) on the outcomes in each exercise protocol. Two‐way repeated measures ANOVA was used to assess the effect of protocol (RUN_100%RCP_, RUN_130%RCP_, and RUN_150%RCP_) and time×protocol interactions on the outcomes. Assumptions of sphericity were explored and controlled for all variables with the Greenhouse–Geisser adjustment, where necessary. If significant main effects or interactions were observed, a Bonferroni post hoc pairwise comparison was performed. Partial eta squared ($\eta_{p}^{2}$) was calculated to estimate effect sizes, with values of 0.01, 0.06, and 0.14 representing small, moderate, and large effects, respectively.

## 3 Results

Maximal and submaximal responses recorded during the GXT are displayed in Table 1. RUN_100%RCP_ was performed at 12.1 ± 0.9 km/h while RUN_130%RCP_ and RUN_150%RCP_ were performed at 15.7 ± 1.2 and 18.3 ± 1.4 km/h, respectively. These intensities corresponded to 75 ± 5%, 98 ± 7% and 113 ± 8% of v$\dot{V}O_{2\;\max}$, respectively. Absolute and relative intensities were lower in RUN_100%RCP_ than in both RUN_130%RCP_ and RUN_150%RCP_; with RUN_130%RCP_ also presenting lower absolute and relative intensities than RUN_150%RCP_ (F_2,22_ > 1914.01, *p* < 0.01, $n_{p}^{2}$ > 0.99, all *post‐hoc p* < 0.01). The estimated distance covered by the participants in each protocol was 8.1 ± 0.6 km.

**TABLE 1:** Maximal and submaximal physiological parameters measured during GXT.

Parameter	Mean ± SD	(CI 95%)
$\dot{V}O_{2\;\max}$ (L/min)	3.70 ± 0.39	(3.45–3.95)
$\dot{V}O_{2\;\max}$ (mL/kg/min)	52.2 ± 6.9	(47.8–56.5)
v$\dot{V}O_{2\;\max}$ (km/h)	16.1 ± 1.5	(15.1–16.9)
RCP		
$\dot{V}O_{2}$ (L/min)	3.08 ± 0.35	(2.85–3.29)
$\dot{V}O_{2}$ relative to $\dot{V}O_{2\;\max}$ (%)	83 ± 8	(78–88)
Velocity (km/h)	12.1 ± 0.9	(11.5–12.7)
Velocity relative to $\dot{V}O_{2\;\max}$ (%)	75 ± 5	(72–78)

*Note:* Data are presented as mean ± standard deviation (confidence interval of 95%).

Abbreviations: $\dot{V}O_{2}$: oxygen consumption; $\dot{V}O_{2\;\max}$: maximal oxygen consumption; v$\dot{V}O_{2\;\max}$: velocity associated to $\dot{V}O_{2\;\max}$; RCP: respiratory compensation point.

### 3.1 Metabolic, Respiratory, and Perceptual Responses During RUN_100%RCP_, RUN_130%RCP_, and RUN_150%RCP_

**FIGURE 2:**
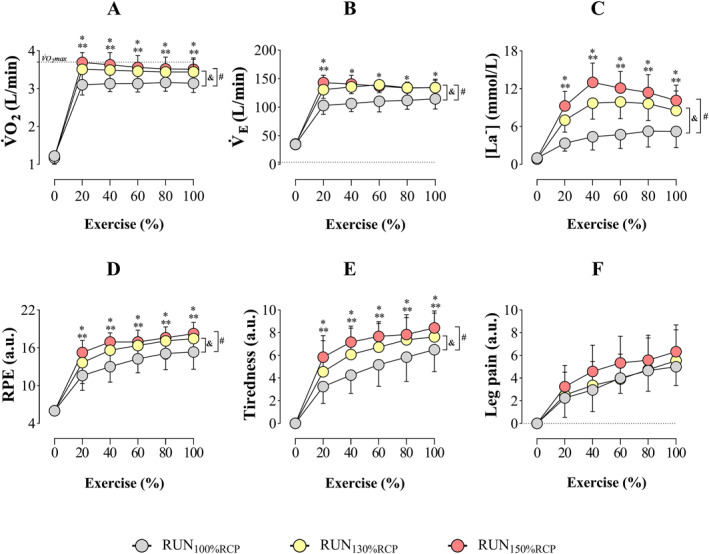
Responses throughout running protocols. $\dot{V}O_{2}$, rate of oxygen consumption; [La^−^], blood lactate concentration; RPE, rating of perceived exertion. Statistical results were determined by a two‐way (time and running protocol) repeated measures analysis of variance (ANOVA); &, RUN_100%RCP_ different from RUN_130%RCP_ (main effect of running protocol, *post‐hoc p* < 0.05); #, RUN_100%RCP_ different from RUN_150%RCP_ (main effect of running protocol, *post‐hoc p* < 0.05); *, RUN_100%RCP_ different from RUN_150%RCP_ at this time point (time×protocol effect, *post‐hoc p* < 0.05); **, RUN_100%RCP_ different from RUN_130%RCP_ at this time point (time × protocol effect, *post‐hoc p* < 0.05); Statistical results from one‐way ANOVA, which assessed main effect of time in each exercise protocol, are described in the *results* section and are not shown in the figure to improve the clarity of the graphs; *N* = 12.

Physiological and perceptual responses measured during running trials are displayed in Figure 2 (A‐I). There were main effects of time (F_5,55_ > 28.43, *p* < 0.01, $n_{p}^{2}$ > 0.72) for $\dot{V}O_{2}$, ${\dot{V}}_{E}$, [La^−^], RPE, tiredness, and leg pain. During all protocols, these outcomes increased from warm‐up to 20%–40% of exercise completion (all *post‐hoc p* < 0.01). A main effect of running protocol (F_2,22_ > 18.17, *p* < 0.01, $n_{p}^{2}$ > 0.62) was evidenced for $\dot{V}O_{2}$, ${\dot{V}}_{E}$, [La^−^], RPE, and tiredness. $\dot{V}O_{2}$, ${\dot{V}}_{E}$, [La_−_], RPE, and tiredness were lower during RUN_100%RCP_ trial compared to both RUN_130%RCP_ and RUN_150%RCP_ trials (all *post hoc p* < 0.01). A time×protocol interaction (F_10,110_ > 11.80, *p* < 0.01, $n_{p}^{2}$ > 0.51) was evidenced for $\dot{V}O_{2}$, ${\dot{V}}_{E}$, [La^−^], RPE, and tiredness. $\dot{V}O_{2}$, [La^−^], RPE, and tiredness were lower from 20% to 100% of exercise completion in the RUN_100%RCP_ trial compared to both RUN_130%RCP_ and RUN_150%RCP_ trials (all *post hoc p* < 0.01). ${\dot{V}}_{E}$ was lower at 20% of exercise completion in the RUN_100%RCP_ trial compared to both RUN_130%RCP_ and RUN_150%RCP_ (*post hoc p* < 0.01). Additionally, ${\dot{V}}_{E}$ was also lower in the RUN_100%RCP_ trial compared to RUN_150%RCP_ trial from 40% to 100% of exercise completion.

### 3.2 Neuromuscular Function After RUN_100%RCP_, RUN_130%RCP_, and RUN_150%RCP_

**FIGURE 3:**
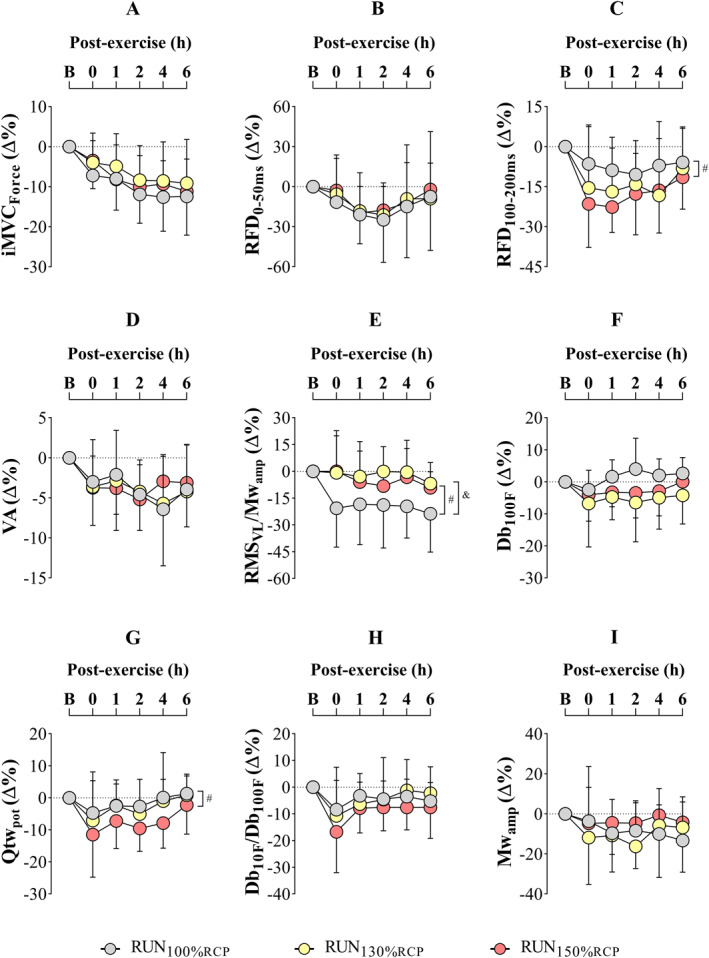
Markers of global, central, and peripheral factors underlying knee‐extensors neuromuscular function. iMVC_Force_, highest voluntary force recorded during maximal voluntary isometric contractions of knee‐extensors; RFD_0 – 50 ms_: early rate of force development (0–50 ms); RFD_100 – 200 ms_: late rate of force development (0–50 ms); VA, voluntary activation measured through twitch interpolation technique; RMS_VL_/Mw_amp_, ratio between the root mean square of electromyography signal from *vastus lateralis* during iMVC (0.5‐s around iMVC_Force_) and peak‐to‐peak M‐wave amplitude (Mw_amp_); Qtw_pot_, force evoked by a potentiated single stimulus at relaxed knee‐extensors; Db100_F,_ force evoked by a supramaximal high‐frequency (100 Hz) stimulus at relaxed knee‐extensors; Db10_F_/Db100_F_, ratio between the force evoked by a supramaximal high‐frequency (100 Hz) and low‐frequency (10 Hz) doublet stimulus at relaxed knee‐extensors. Statistical reports come from a two‐way (time and running protocol) repeated measures analysis of variance (ANOVA); &, RUN_100%RCP_ different from RUN_130%RCP_ (main effect of running protocol, *post‐hoc p* < 0.05); #, RUN_100%RCP_ different from RUN_150%RCP_ (main effect of running protocol, *post‐hoc p* < 0.05); Statistical results from one‐way ANOVA, which assessed main effect of time in each exercise protocol, are described in the *results* section and are not shown in the figure to improve the clarity of the graphs; *N* = 12.

The changes in neuromuscular function after each running protocol are displayed in Figure 3 (A‐I). For all running protocols, main effects of time were observed for iMVC_Force_ (F_5,55_ > 13.71, *p* < 0.01, $n_{p}^{2}$ > 0.55) and VA (F_5,55_ > 6.12, *p* < 0.01, $n_{p}^{2}$ > 0.36). Specifically, iMVC_Force_ remained depressed for at least 6 h after exercises (all *post‐hoc p* ≤ 0.01 in all running protocols), whereas VA reduction persisted for up to 4 h post‐exercise (baseline vs. 6 h: *post‐hoc p* > 0.05 in all running protocols). In RUN_100%RCP,_ a main effect of time was evidenced for RMS_VL_/Mw_amp_ (F_5,55_ = 7.84, *p* = 0.01, $n_{p}^{2}$ = 0.41), indicating that it remained depressed for at least 6 h post‐exercise (all *post‐hoc p* ≤ 0.01). In RUN_130%RCP_, main effects of time were observed for RFD_100–200 ms_ (F_5,55_ = 3.20, *p* = 0.01, $n_{p}^{2}$ = 0.22) and Mw_amp_ (F_5,55_ = 2.60, *p* = 0.03, $n_{p}^{2}$ = 0.19); both were reduced at 1 and 2 h post‐exercise (all *post‐hoc p* ≤ 0.02). In RUN_150%RCP_, main effects of time were observed for RFD_100–200 ms_ (F_5,55_ = 3.20, *p* = 0.02, $n_{p}^{2}$ = 0.22), Qtw_pot_ (F_5,55_ = 4.26, *p* = 0.01, $n_{p}^{2}$ = 0.28), and RMS_VL_/Mw_amp_ (F_5,55_ = 2.41, *p* = 0.04, $n_{p}^{2}$ = 0.18). Specifically, RFD_100–200 ms_ and Qtw_pot_ remained decreased for at least 4 and 6 h post‐exercise, respectively, while RMS_VL_/Mw_amp_ decreased at 2 and 4 h following exercise (all *post‐hoc p* ≤ 0.03). A main effect of running protocol was observed for RMS_VL_/Mw_amp_ (F_2,22_ = 9.45, *p* < 0.01, $n_{p}^{2}$ = 0.46), RFD_100–200 ms_ (F_2,22_ = 3.71, *p* < 0.01, $n_{p}^{2}$ = 0.25) and Qtw_pot_ (F_2,22_ = 5.06, *p* = 0.02, $n_{p}^{2}$ = 0.31). The RMS_VL_/Mw_amp_ presented greater reduction following RUN_100%RCP_ compared to both RUN_130%RCP_ and RUN_150%RCP_ (all *post‐hoc p* < 0.03), while RFD_100–200 ms_ and Qtw_pot_ presented greater reduction following RUN_150%RCP_ compared to RUN_100%RCP_. There was no time or running protocol effects on Db100_F_ or Mw_amp_ (*p* > 0.07, $n_{p}^{2}$ < 0.23). There was no time × protocol interaction for any marker of neuromuscular function (*p* > 0.18, $n_{p}^{2}$ < 0.11).

### 3.3 Perceived Fatigability After RUN_100%RCP_, RUN_130%RCP_, and RUN_150%RCP_

**FIGURE 4:**
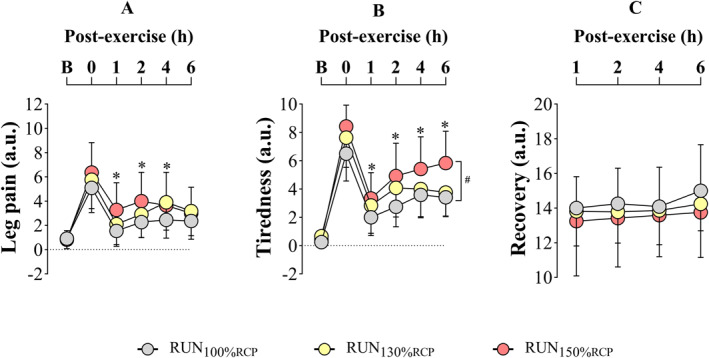
Perceived fatigability responses. Statistical reports come from a two‐way (time and protocol) repeated measures analysis of variance (ANOVA); #, RUN_100%RCP_ different from RUN_150%RCP_ (main effect of running protocol, *post‐hoc p* < 0.05); *, RUN_100%RCP_ different from RUN_150%RCP_ at this time point (time×protocol effect, *post‐hoc p* < 0.05); Statistical results from one‐way ANOVA, which assessed main effect of time in each exercise protocol, are described in the *results* section and are not shown in the figure to improve the clarity of the graphs; *N* = 12.

The changes in perceived fatigability are presented in Figure 4 (A‐C). For all running protocols, main effects of time were observed for leg pain (F_4,44_ > 10.34, *p* < 0.01, $n_{p}^{2}$ > 0.49; Figure 4A) and tiredness (F_4,44_ > 11.28, *p* < 0.01, $n_{p}^{2}$ > 0.51; Figure 4B), whereby both persisted greater than baseline for at least 6 h post‐exercise (all post hoc *p* ≤ 0.01 in all running protocols). There was also a main effect of running protocol for tiredness (F_2,22_ = 8.70, *p* = 0.02, $n_{p}^{2}$ = 0.44), whereby tiredness was higher following RUN_150%RCP_ compared to RUN_100%RCP_ (all post hoc *p* ≤ 0.01). Significant time×protocol interactions were observed for tiredness (F_8,88_ = 3.17, *p* < 0.01, $n_{p}^{2}$ = 0.22) and leg pain (F_8,88_ > 2.63, *p* < 0.05, $n_{p}^{2}$ = 0.19), whereby tiredness and leg pain were greater for at least 6 h and up to 4 h, respectively, following RUN_150%RCP_ compared to RUN_100%RCP_ (all *post‐hoc p* ≤ 0.01). There were no effects of time, running protocol, or interaction for perceived recovery (*p* > 0.65; Figure 4C).

## 4 Discussion

This study investigated neuromuscular function and perceived fatigability after distinct high‐intensity endurance running protocols of matched volume (8.1 ± 0.6 km). There was no difference for knee‐extensor function markers in specific time points after exercise, but aggregate data over 6 h revealed that variations in the exercise protocol led to differences in the central and peripheral factors underlying neuromuscular fatigue. Specifically, RUN_100%RCP_ induced greater reductions in neural drive to *vastus lateralis* (RMS_VL_/Mw_amp_) compared to both RUN_130%RCP_ and RUN_150%RCP_, while RUN_150%RCP_ caused greater impairments in knee‐extensor contractile function (reduction in RFD_100–200 ms_ and Qtw_pot_) compared to RUN_100%RCP_. Significant time×protocol interactions showed that leg pain and tiredness were exacerbated for up to 4 h and for at least 6 h, respectively, after RUN_150%RCP_ compared to RUN_100%RCP_. To our knowledge, this is the first study to demonstrate that the characteristics and extent of neuromuscular fatigue and perceived fatigability in the hours following high‐intensity endurance running are dependent on the exercise protocol.

### 4.1 Neuromuscular Function Impairments After RUN_100%RCP_, RUN_130%RCP_, and RUN_150%RCP_: Maximal Force‐Generating Capacity of the Knee‐Extensors

The current study evidenced a similar impairment on voluntary peak force of knee‐extensor across running protocols (aggregate reduction of *−*10.1% at 2–6 h post‐exercise; Figure 3A). This degree of force loss is greater than reported by Skof and Strojnik after a 5 × 300 m track run (*−*4%) (2006b) and after a 6 km track run (*−*6%) (2006a). However, it is lower than those reported by Brownstein et al. (2022) after 17 km treadmill running at a velocity 5% above gas‐exchange threshold (*−*14%) and Ross et al. (2010) after 20 km treadmill time‐trial running (*−*17%). The similar reduction in maximal force across all running protocols in the current study suggests that once the same distance has been covered, variations in running protocol do not affect the extent of loss of knee extensors’ isometric peak force. Notably, in cycling, MacDougall et al. (2024) evidenced greater reductions in knee extensors’ isometric peak force after 10 × 2 min effort at 80% of peak power output (−27%), compared to 38 min continuous effort at 54% of peak power output (−15%). Such difference was evidenced even with very similar exercise volume between trials (i.e., 293 kJ vs. 296 kJ). The discrepancies between our findings and those reported by MacDougall et al. (2024), likely stem from differences in neuromuscular and metabolic demands between running and cycling (Bijker et al. 2002; Brownstein et al. 2022; Tomazin et al. 2017). Although we cannot fully explain this difference, we speculate that the likely more forceful eccentric contractions during the stance phase of RUN_150%RCP_ (Wiewelhove et al. 2015) may have induced greater impairments in muscle contractile properties, leading to the more pronounced reduction in late RFD (Maffiuletti et al. 2016). However, these mechanisms warrant further investigation.

### 4.2 Neuromuscular Function Impairments After RUN_100%RCP_, RUN_130%RCP_, and RUN_150%RCP_: Peripheral Factors

The aggregate reduction in Qtw_pot_ 6 h after exercise was more pronounced following RUN_150%RCP_ (−8%) compared to RUN_100%RCP_ (−2%) (Figure 3G). These findings extend previous observations from cycle‐based efforts (Iannetta et al. 2022; Azevedo et al. 2022), demonstrating that increased running intensity exacerbates impairments in muscle contractile function. Furthermore, the aggregate reduction in the late RFD phase was greater after RUN_150%RCP_ (−18%) than RUN_100%RCP_ (−8%; main effect of protocol, *p* < 0.05; Figure 3C). Because early and late RFD phases heavily depend on neural and muscle properties, respectively (Maffiuletti et al. 2016; D’Emanuele et al. 2021), our data suggest that variations in running protocol, such as increases in intensity, may influence muscle contractile properties rather than the neural factors underpinning the RFD. Notably, although metabolic responses differed between RUN_100%RCP_ and RUN_130%RCP_ (mean [La^−^] of ∼ 4.6 mmol/L vs. 9.0 mmol/L, respectively; mean $\dot{V}O_{2}$ of 3.1 L/min vs. 3.5 L/min, respectively), this difference did not lead to distinct levels of impairment in knee‐extensors contractile function. In addition to the greater accumulation of metabolites (e.g., [Pi], [H^+^], [K^+^], [La^−^]) associated with impaired muscle contractile function during exercise at intensities far above MMSS (Chidnok et al. 2013), increases in running velocity may also exacerbate muscle fiber disruption induced by mechanical loading and microtrauma (Stocchero et al. 2014; Wiewelhove et al. 2015). The combined influence of intensity on muscle microtrauma and metabolic disturbances likely contributes to the exacerbated knee‐extensors contractile function as intensity increases. Future research should assess the interactions of increasing exercise intensity on metabolic disturbances, muscle microtrauma, and impairments in knee‐extensors contractile function following high‐intensity endurance running.

### 4.3 Neuromuscular Function Impairments After RUN_100%RCP_, RUN_130%RCP_, and RUN_150%RCP_: Central Factors

Changes in central factors related to neuromuscular function were assessed using two measures in the current study: VA and RMS_VL_/Mw_amp_. We observed similar reductions in VA across running protocols (−4%), which persisted for at least 4 h after all exercises (main effects of time, *p* < 0.05; Figure 3D). The aggregate level of reduction in VA evidenced in the current study exceeded that reported after 6 km and 5 × 300 m track runs (both −1%) (Skof and Strojnik 2006a, 2006b). However, it was lower than the reductions induced by a 17 km treadmill running at a velocity 5% above gas‐exchange threshold (−16%) and by a 20 km treadmill time‐trial running (−12%). Our findings extend to running the conclusions of Iannetta et al. (2022), on similar VA reductions after cycling until task failure at intensities below 120% of peak power output. Yet, the lack of an intensity‐dependent effect of locomotor exercises on VA remains debated (Thomas et al. 2016, 2015). Despite the similar reductions in VA across running protocols, we observed a greater reduction in RMS_VL_/Mw_amp_ following RUN_100%RCP_ (−24%) compared to both RUN_130%RCP_ (−3%) and RUN_150%RCP_ (−6%) ‒ main effect of protocol, *p* < 0.05; Figure 3E. While VA, measured by the twitch interpolation technique is a key metric, RMS_VL_/Mw_amp_ is also used to assess changes in motor drive output, along with other measures like central activation ratio and the comparison of maximal voluntary force to force evoked by high‐frequency tetanus (Millet et al. 2011). Although caution is warranted when interpreting bipolar EMG signals due technical concerns (Farina et al. 2010), previous research evidenced that RMS_VL_/Mw_amp_ was more sensitive than VA in detecting changes in motor drive output during scenarios with low metabolic disturbance, such as passive stretch (Avela et al. 1999; Trajano et al. 2013) and jogging (Racinais et al. 2007). Accordingly, our findings suggest a more pronounced decline in neural drive to the *vastus lateralis* after RUN_100%RCP_ compared to the other protocols (Racinais et al. 2007). Given the lower metabolic demand during RUN_100%RCP_ (mean [La^−^] and $\dot{V}O_{2}$ of ∼ 4.6 mmol/L and 3.1 L/min) compared to RUN_130%RCP_ (9.0 mmol/L and 3.5 L/min) and RUN_150%RCP_ (11.2 mmol/L and 3.6 L/min), differences in inhibitory feedback from group III/IV afferents to the central nervous system (Amann et al. 2011) are unlikely to explain the observed differences in RMS_VL_/Mw_amp_ between the protocols. Unfortunately, our data preclude a full understanding of the mechanisms underpinning this finding. Thus, future studies should employ measures like motor evoked potentials, cervicomedullary motor evoked potentials, Hoffman reflex, and motor unit firing rate to further elucidate the effects of high‐intensity endurance running on neuromuscular function at the spinal and supraspinal levels (Millet et al. 2011; Zero and Rice 2021).

### 4.4 Perceived Fatigability Responses After RUN_100%RCP_, RUN_130%RCP_, and RUN_150%RCP_

Perceived recovery remained suboptimal and unchanged for at least 6 h after all running protocols (no main effects of time, running protocol, or interactions, *p* > 0.05, Figure 4C). Participants also reported increased tiredness and leg pain for at least 6 h after all running protocols (main effects of time, *p* < 0.05; Figure 4A and 4B). However, tiredness and leg pain were exacerbated following RUN_150%RCP_ compared to RUN_100%RCP_ (time×protocol interaction, *p* < 0.05; Figures 4A and 4B). The elevated leg pain following RUN_150%RCP_ extend to running the previously observed intensity‐dependent effect on leg pain when cycling far above MMSS (Iannetta et al. 2022; MacDougall et al. 2024). This effect seem to be linked to the greater metabolite accumulation in the prime mover muscles when exercising at these intensities (Iannetta et al. 2022; Pollak et al. 2014). Tiredness was also greater after RUN_150%RCP_ compared to RUN_100%RCP_. As demonstrated by Iannetta et al. (2022), in cycling, tiredness at task failure increases as exercise duration extends. Due to the inclusion of rest intervals, RUN_150%RCP_ was the longest trial in the current study (∼80 min, ∼70 min, and ∼42 min for RUN_150%RCP,_ RUN_130%RCP_, and RUN_100%RCP_, respectively). Previous research suggests that sustain high‐intensity efforts may lead to changes in the concentration of neurotransmitters and metabolites in specific regions of the brain (Behrens et al. 2023; Duclos and Tabarin 2016). These changes are thought to promote tiredness, lethargy, and sleepiness during and after exercise (i.e., cognitive performance and perceived fatigue) (Behrens et al. 2023; Duclos and Tabarin 2016). Therefore, despite matched distances, the longer duration (including rest intervals) of RUN_150%RCP_ likely contributed to exacerbating tiredness sensation post‐exercise compared to RUN_100%RCP_.

### 4.5 Experimental Considerations

There was a delay of 57 ± 9 s between the start of the neuromuscular assessments and the end of the exercise session. This may have led to an underestimation of the level of neuromuscular function impairment evidenced immediately after exercise. Although there are setups to assess neuromuscular function immediately after cycling (Iannetta et al. 2022), such methodological apparatus is difficult to implement for treadmill running and appears to be an inherent flaw when investigating more ecological locomotor exercises. Nevertheless, recovery of neuromuscular outcomes has been suggested to be up to 2% (not statistically significant) in the first minute after locomotor exercises (Krüger, Aboodarda, Jaimes, Samozino, et al. 2019). Therefore, it is likely that this delay had a negligible influence on the findings and conclusions of the current study. Furthermore, a discrepancy was observed between RMS_VL_/Mw_amp_ and VA outcomes (both reduced after exercise, but only RMS_VL_/Mw_amp_ showed a main effect of running protocol). Although we are unable to fully explain this discrepancy, it had also been observed by other studies, specifically in scenarios with low metabolic disturbance (Avela et al. 1999; Trajano et al. 2013; Racinais et al. 2007). Given that RMS_VL_/Mw_amp_ is also extensively used to reflect changes in the level of motor drive output (Millet et al. 2011; Millet and Lepers 2004), we are confident in the reported effect of running protocol on central factors underlying neuromuscular function. Notably, although RMS_VL_/Mw_amp_ demonstrated a greater decrease following RUN_100%RCP_, the reduction in voluntary peak force of the knee‐extensors was similar across all running protocols. We are unable to fully explain this finding. We speculate that the relatively preserved contractile function observed after RUN_100%RCP_ may have compensated for the greater reductions in neural drive, thereby maintaining post‐exercise voluntary peak force at levels similar to the other protocols. Alternatively, the current study may have been underpowered to detect differences in voluntary peak force between protocols. Future research with a larger sample size is warranted to elucidate how the substantial reductions in neural drive following RUN_100%RCP_ influence post‐exercise voluntary peak force. Moreover, we did not assess other muscle groups, such as plantar flexors, despite their important role in running (Millet and Lepers 2004), or included tests involving dynamic contractions, which are known to be more sensitive to exercise‐induced fatigability (Krüger, Aboodarda, Jaimes, MacIntosh, et al. 2019). We assessed knee‐extensors function using isometric contractions due to electrical stimulation procedures and the widespread use of isometric contraction assessments in neuromuscular research, enhancing reproducibility and facilitating cross‐study comparisons. Additionally, our study included only male participants. Since existing evidence suggests lower fatigability in females compared with males (Azevedo et al. 2022), future research should assess the interactions of sex on the dynamics of performance and perceived fatigability response after high‐intensity endurance running.

## 5 Conclusion and Perspective

Maximal isometric knee‐extensors force, voluntary activation (measured by twitch interpolation technique), and perceived recovery remained similarly impaired for at least 6 h following RUN_100%RCP_, RUN_130%RCP,_ and RUN_150%RCP_. However, the aggregate data until 6 h post‐exercise evidenced that *vastus lateralis* neural drive (RMS_VL_/Mw_amp_) was more reduced after RUN_100%RCP_ compared to RUN_130%RCP_ and RUN_150%RCP_; and impairments in knee‐extensors contractile function (reduced RFD_100–200 ms_ and Qtw_pot_) were greater following RUN_150%RCP_ compared to RUN_100%RCP._ Leg pain and tiredness were also greater for up to 4 h and for at least 6 h, respectively, after RUN_150%RCP_ compared to RUN_100%RCP_. These novel findings demonstrate that underlying aspects of neuromuscular fatigue and perceived fatigability in the hours following high‐intensity endurance running depend on exercise protocol. This knowledge may be particularly useful for optimizing short‐term recovery management in demanding schedules, such as those involving repeated running efforts or additional sessions within a single day.

## Conflicts of Interest

The authors declare no conflicts of interest.

## Data Availability

The data that support the findings of this study are available from the corresponding author upon reasonable request.
